# Zooid arrangement and colony growth in *Porpita porpita*

**DOI:** 10.1186/s12983-025-00565-3

**Published:** 2025-06-23

**Authors:** Kohei Oguchi, Akiteru Maeno, Keita Yoshida, Gaku Yamamoto, Hisanori Kohtsuka, Casey W. Dunn

**Affiliations:** 1 Misaki Marine Biological Station, The University of Tokyo, Miura, Kanagawa 238-0225 Japan; 2 Department of Biology, Undergraduate School of Biological Sciences, Tokai University, Sapporo, Hokkaido 005-8601 Japan; 3 Cell Architecture Laboratory, National Institute of Genetics, Yata 1111, Mishima, Shizuoka 411-8540 Japan; 4 Enoshima Aquarium, Katasekaigan, Fujisawa, Kanagawa 251-0035 Japan; 5 Department of Ecology and Evolutionary Biology, Curator of Invertebrate Zoology, Peabody Museum, Yale University, 170 Whitney Ave, New Haven, CT 06511 USA

**Keywords:** Porpita, Coloniality, Zooids, Micro-CT, Histology

## Abstract

**Background:**

The blue button, *Porpita porpita* (Porpitidae), is a highly integrated colonial animal—i.e., a superorganism. It has multiple genetically identical bodies (zooids) that arise from the same embryo and are functionally specialized for distinct tasks and arranged in precise patterns. Their colonies include a float, coenosarc, gastrozooid (feeding polyp), gonozooids (reproductive polyps), and dactylozooids (tentacle polyp). Colonies are fragile and difficult to culture, leaving much about their development and lifecycle unknown. We provide new insight into colony development of *P. porpita* with morphological observation and histological analysis using histological sections and micro-CT technology.

**Results:**

From 2019 to 2024, we collected over 267 *P. porpita* specimens of varying sizes to study colony development. Morphological investigation revealed that the number and length of gastrozooids, gonozooids and dactylozooids increased with float size. Further observation by histological section and micro-CT technique revealed the internal structures of colonies, including gastrozooid, floats, and aboral chambers that connect various zooids. Immature gonozooids and dactylozooids were observed near mature ones, providing insight into their colony level development. In addition, some colonies showed irregular shapes, but still contained at least one gastrozooid, illustrating the structural variation within the species.

**Conclusions:**

Our study revealed that gonozooids and dactylozooids increased in both number and size as the colony develops. Moreover, the growth zones for dactylozooids are located at the boundary of the mantle and coenosarc, and gonozooids emerge along the entire epithelium between the gastrozooid and dactylozooids. Colony growth generally follows a pattern proportional to colony circumference and area, and some colonies show irregular shapes, suggesting they have high regenerative capabilities. Taken together, these findings enhance our understanding of the ecology and life history of *P. porpita*.

**Supplementary Information:**

The online version contains supplementary material available at 10.1186/s12983-025-00565-3.

## Background

Colonial animals, such as those in the Cnidaria, Bryozoa, Entoprocta, and Chordata, consist of species in which zooids (individual units) grow via asexual reproduction alongside each other. These zooids are homologous to the entire body of solitary free-living organisms. In such colonies, individual boundaries can be unclear, and zooids can form highly complex and specialized colonial types [1–4]. Colonies of such animals are sometimes called “super-organisms” since—despite being composed of many individual bodies—they behave as if they were a single individual due to their division of labor and physiological integration [5–8]. Coloniality is found in various animal groups, but colonies in Cnidaria, especially those belonging to Porpitidae and Siphonophora, are key representatives of “super-organisms” whose sophisticated colonies of zooids behave as a single individual [5–8].

Porpitidae (Hydrozoa: Capitata) contains only two known species, *Porpita porpita* and *Velella velella*, and their worldwide genetic diversity and the existence of cryptic species are not clear [9–12]. Both are pleuston species that are widely spread throughout the world, as they drift on the surface of the ocean by creating floats made of chitin [13–18]. Although their overall structure is very similar, the colonies of *V. velella* are elliptical with transparent sails, whereas the colonies of *P. porpita* are circular and lack sails [13–17]. In general, a colony consists of a float, a coenosarc, and multiple functionally and morphologically specialized polyps [e.g., 19]. The feeding polyp, termed the “gastrozooid,” is located in the lower center of the float, where it is surrounded by “gonozooids” that release medusa that then reproduce sexually, as well as “dactylozooids” that behave like tentacles to capture prey at the edge of the colony. Such colonies are believed to have evolved from benthic or epibiotic ancestors in the Capitata lineage [12]. In many of these ancestral species, the polyps form asexual generations connected by stolon network and undergo irregular budding [20]. Thus, it is hypothesized that the functional specialization of zooids and their spatially organized arrangement contribute to the formation of sophisticated colonies, such as those seen in *Porpita* and *Velella*. These colonies are fragile and are difficult to culture in the lab. To date, little is known regarding their life cycle, zooid arrangement, and colony development.

Compared to the scarce research on *P. porpita* and *V. velella*, considerable progress has been made in recent years studying siphonophore colony formation [21–23]. For example, in *Nanomia septata* [24], a species of siphonophore, there are two growth zones on the linearly extending “stem.” One is the “nectosomal growth zone” which produces nectophores (i.e., swimming zooids) and the other is the “siphosomal growth zone” which produces gastrozooids (feeding polyps), palpons (digestive polyps), and gonophores [6, 21, 22]. In both growth zones, stem cells are localized and the positioning of specific zooids in specific places permits the creation of a precise spatial structure of zooid morphologies and functions, thereby comprising a highly sophisticated colony [22]. On the other hand, the colony structure of *P. porpita* differs strongly from siphonophores, since the mode of zooid budding is expected to be different, its growth zone is unclear, and the corresponding mechanism of colony development in *P. porpita* also remains unclear.

To better elucidate the mechanisms responsible for colony development in *P. porpita*, in this study we performed morphological observation as well as histological observation using classical sectioning and microfocus X-ray computed tomography (micro-CT). Micro-CT is a technology that uses X-rays to observe structures, including internal colony structure. It is also capable of noninvasively scanning the cross sections of an object in order to construct three-dimensional structures. Recently, micro-CT systems have become a powerful tool for morphological observation in a wide variety of biological studies [25], and therefore in this study we applied this technique to determine the colony structure of *P. porpita*. Furthermore, we examined several heteromorphic colonies found along the coast of Sagami Bay (Kanagawa, Japan) and discuss the high regenerative capabilities of these colonies. By combining these data sources, this paper sheds insight into the developmental pattern and life cycle of colonies of *P. porpita*.

## Methods

### Sample collection and morphological observation

Samples of *P. porpita* were found along the beaches and rocky shores of Kanagawa prefecture (Japan) in 2019–2024 and were collected in plastic bags. After collection, specimens were fixed in 4% paraformaldehyde (PFA) in PBS or FAA solution (ethanol: formalin: acetic acid = 16: 6: 1) then dissected using fine tweezers under a stereomicroscope SZX16 (Olympus, Tokyo, Japan). The number of gonozooids, dactylozooids, and gastrozooids were counted for each specimen. For large colonies (i.e., those colonies with floats larger than 14 mm in diameter), we determined the number of gonozooids and dactylozooids by counting them in only one quarter of the colony, then estimating the number for the whole colony by multiplying by four. We also measured float diameter, maximum gonozooid and dactylozooids length, and the width of gastrozooids.

### Histological observation by histological section

To investigate histological features, we made paraffin sections of whole colonies. First, we fixed colonies in 4% PFA for 24 h, then preserved specimens in 70% EtOH until further use. Samples were then dehydrated in increasing concentrations of ethanol, transferred into xylene, and finally embedded in paraffin. Serial Sects. (8 μm thick) in horizontal planes were then prepared using a microtome (Spencer Lens, Buffalo, NY) and the resulting sections were then stained with hematoxylin and eosin (HE) staining. Tissue samples on slides were observed using an optical microscope (BX51, Olympus, Tokyo, Japan), and photographs were taken using a digital camera (DP74, Olympus, Tokyo, Japan) attached to the microscope.

Based on studies of siphonophores that form similar colonies [26], the orientation within the colony of *P. porpita* was defined as follows. The side of the gastrozooid bearing the mouth was designated as the oral side, with the opposite side defined as aboral. Similarly, the central region of the float was defined as proximal, with the direction extending away from it designated as distal.

### Histological observation by microfocus X-ray computed tomography (micro-CT)

We conducted further histological observations using a micro-CT scanning protocol described by Maeno et al. [25] Fixation, staining, and scanning were performed under the conditions detailed in Supplementary Table (Additional file 1: Supplementary Table S1)). Briefly, prior to scanning, fixed specimens were pre-stained with 1% phosphotungstic acid (PTA) solution in 70% ethanol or a 1:4 mixture of Lugol's solution and deionized distilled water, as previously described [27, 28]. The samples were scanned using two X-ray microCT instruments, ScanXmate-E090S105 and ScanXmate-CF110TSH320/460 (Comscantechno Co., Ltd. in Yokohama./The current maintenance is being carried out by Voxel Works Co., Ltd. in Tokyo.). Scanned data were reconstructed to tiff format files by coneCTexpress (Comscantechno/Voxel Works). 2D and 3D tomographic images were obtained using OsiriX MD software (Pixmeo, Switzerland). Finally, each video was edited using Premiere Pro (Adobe, Boston, USA).

## Results

### Morphological observations of the colony

**Fig. 1:**
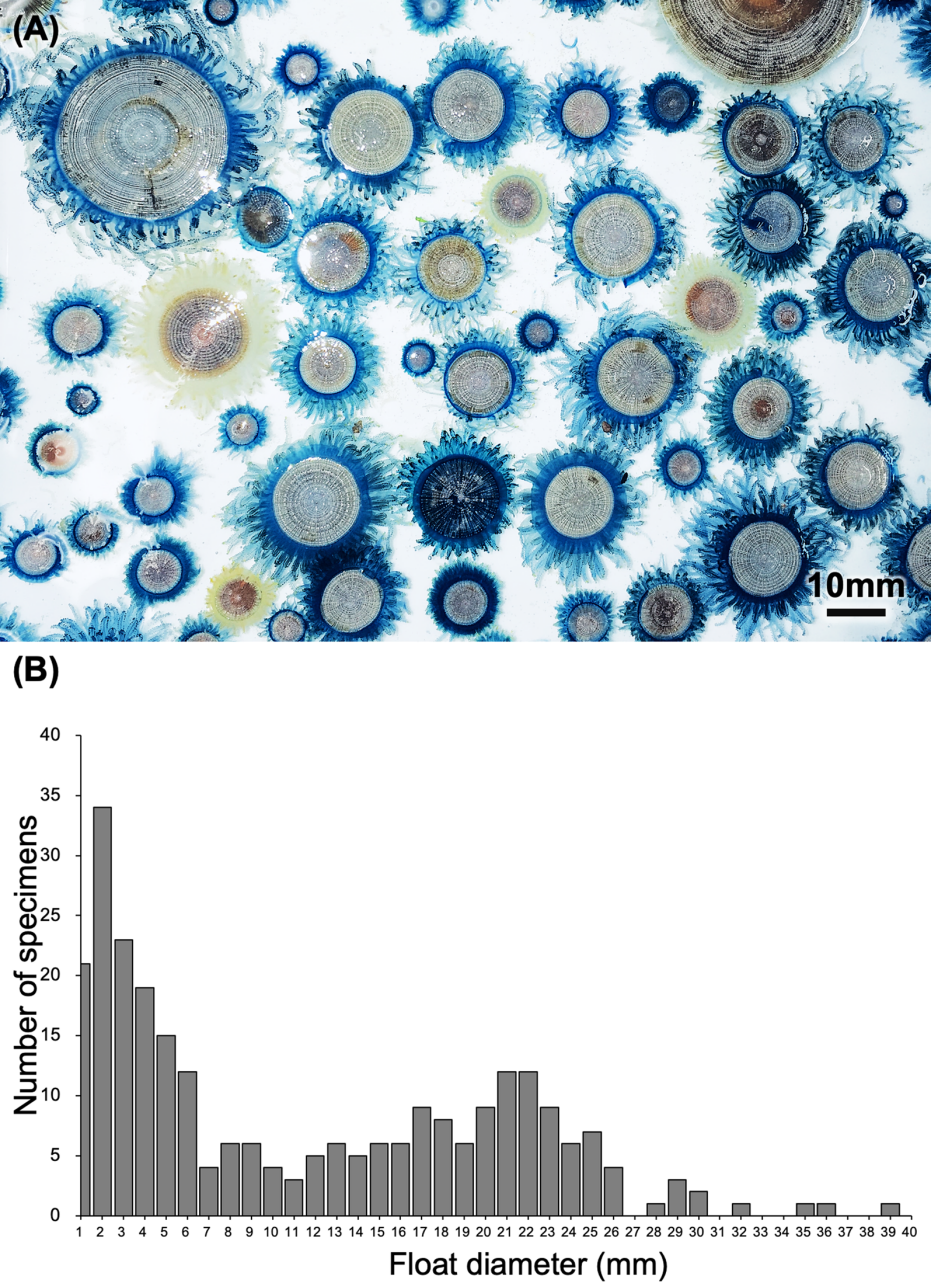
Colonies of *Porpita porpita* of various sizes and colors obtained during the survey (**a**). A histogram showing the frequency of occurrence of each float diameter (**b**)

In total, we conducted surveys from 2019 to 2024 and obtained more than 267 *P. porpita* colony specimens of various sizes and colors in Kanagawa prefecture (Japan) (Fig. 1a). The minimum diameter of the float in obtained specimens was 0.53 mm and the maximum was 38.53 mm (Fig. 1b).

**Fig. 2:**
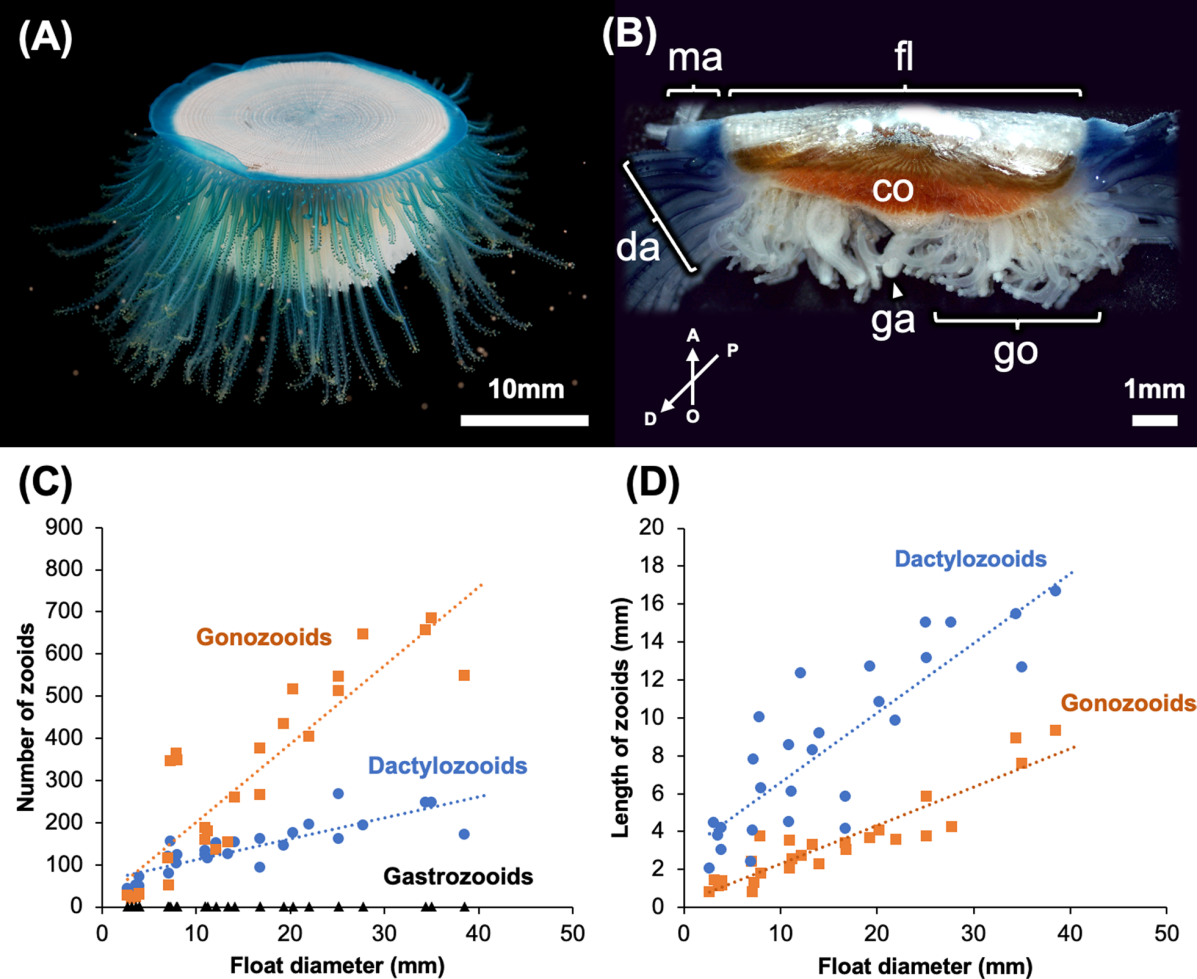
Whole image of a *Porpita porpita* colony and its anatomical structures and characteristics. Whole image of *P. porpita* colony (**a**)*.* Brown dots surrounding the colony are medusae. Fine anatomical structures of the colony as visualized using divided cross sections (**b**). Shown are: mantle (ma), dactylozooids (da), gonozooids (go), gastrozooid (ga), coenosarc (co) and float (fl) (**b**). Arrows indicate the orientation of the colony, a: aboral, o: oral, p: proximal, and d: distal. The number (**c**) and length (**d**) of each structure relative to the size of the float

Next, to investigate the degree of colony development as well as that of several structures such as gonozooids and dactylozooids, we conducted detailed examinations of 27 specimens with nicely preserved colonies (Fig. 2). In general, colonies of *P. porpita* have a float on the aboral side and a structure called a mantle along the edge (Fig. 2a, b). On the oral side of the colony, a gastrozooid is positioned in the center, numerous gonozooids are present surrounding this gastrozooid, and dactylozooids are found on the edges (Fig. 2b). Structurally, we always found this only one gastrozooid regardless of colony size (Fig. 2c); however, the number of gonozooids and dactylozooids tended to increase in proportion to the float size (Fig. 2c). The increase in gonozooid number was higher than that of dactylozooids number, and respective increases could be approximated linearly by the following linear equations: *y* = 18.613x + 15.591 (*R*^2^ = 0.7989) and *y* = 5.0012x + 61.506 (*R*^2^ = 0.7048), respectively. Similarly, the gonozooid and dactylozooids lengths both increased in proportion to the size of the float and these relationships could be approximated by the following linear equations: *y* = 0.2019x + 0.277 (*R*^2^ = 0.854) for gonozooids and *y* = 0.367x + 2.9016 (*R*^2^ = 0.7272) for dactylozooids (Fig. 2d). Unlike gonozooid number, gonozooid length tended to increase proportionately to the size of an increase in dactylozooid length (Fig. 2d). In most cases, medusae were observed on gonozooids, however, no medusae were observed on the gonozooids of colonies with a float size under 10 mm.

### Histological observations

**Fig. 3:**
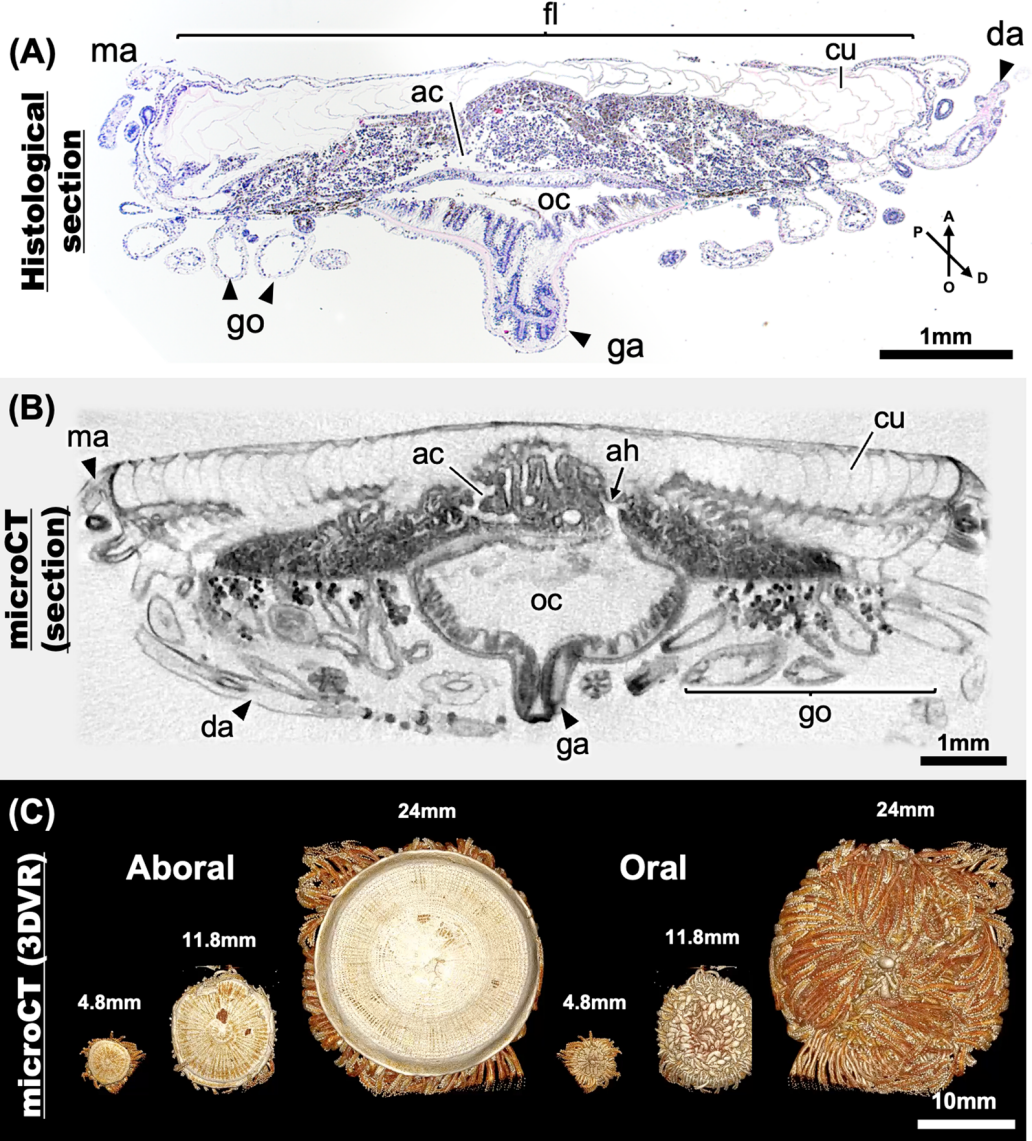
Histology of *Porpita porpita* colonies observed using different methods. Sagittal histological section of a colony (**a**). Sagittal optical section of a colony as determined using micro-CT (**b**). 3D-constructed images of various colony sizes as obtained by micro-CT (**c**). Arrows indicate the orientation of the colony, a: aboral, o: oral, p: proximal, and d: distal. Each structure is shown: mantle (ma), float (fl), cuticle (cu), aboral chamber (ac), aboral hole (ah), oral chamber (oc), dactylozooid (da), gonozooid (go), gastrozooid (ga)

To investigate the histological features of *P. porpita* colonies, we performed both classical sectioning and micro-CT analyses (Fig. 3). Histological sections stained with HE staining were prepared for colonies of a float size of approximately 20 mm (Fig. 3a). For tissue sections sliced in the sagittal plane, a gastrozooid was observed in the center of the oral side, and floats containing several voids were observed on the aboral side, which consisted of a cuticular layer (Fig. 3a). Between the floats and gastrozooid, a coenosarc (centradenia) composed of epithelial cells, a variety of cells of different sizes, nematocysts and a pore space called the aboral chamber were also observed (Fig. 3a; Additional file 2: Supplemental Figure S1). Around the gastrozooid, gonozooids and dactylozooids were observed on the margins (Fig. 3a). As described in previous studies [14, 29], void spaces called “oral chambers” were observed in the gastrozooid, as well as in gonozooids and dactylozooids (Fig. 3a). Micro-CT observation revealed a histological structure that was similar to that observed using histological sections (Fig. 3b; Additional file 3: Supplemental Video S1). We then used obtained section images to create three-dimensional colony structures (Fig. 3c; Additional file 4: Supplemental Video S2). These 3D reconstructed images clearly represent several characteristics of the colony (Fig. 3c; Additional file 4: Supplemental Video S2).

Overall, the gastrozooid appeared as almost pouch-like structure. It was sharply pointed toward the oral side but contained several holes on the aboral side, which was connected to the aboral chamber (Fig. 3b: Additional file 5: Supplemental Video S3; Additional file 6: Supplemental Figure S2). Radial aboral chambers were visible inside the float, where the cuticle extends in concentric circles (Additional file 7: Supplemental Figure S3). The aboral chambers were found to be arranged in mesh-like patterns around the central gastrozooid, but were more regularly arranged along the margin (Additional file 8: Supplemental Figure S4; Additional file 9: Supplemental Video S4). The aboral chamber extended from the center toward the margin and was connected to the gonozooids and dactylozooids (Additional file 10: Supplemental Figure S5). Histological sections also observed these chambers, and revealed a single layer of epithelial cells as well as some cells stained dark with hematoxylin.

### Growth zones in colonies of *P. porpita*

**Fig. 4:**
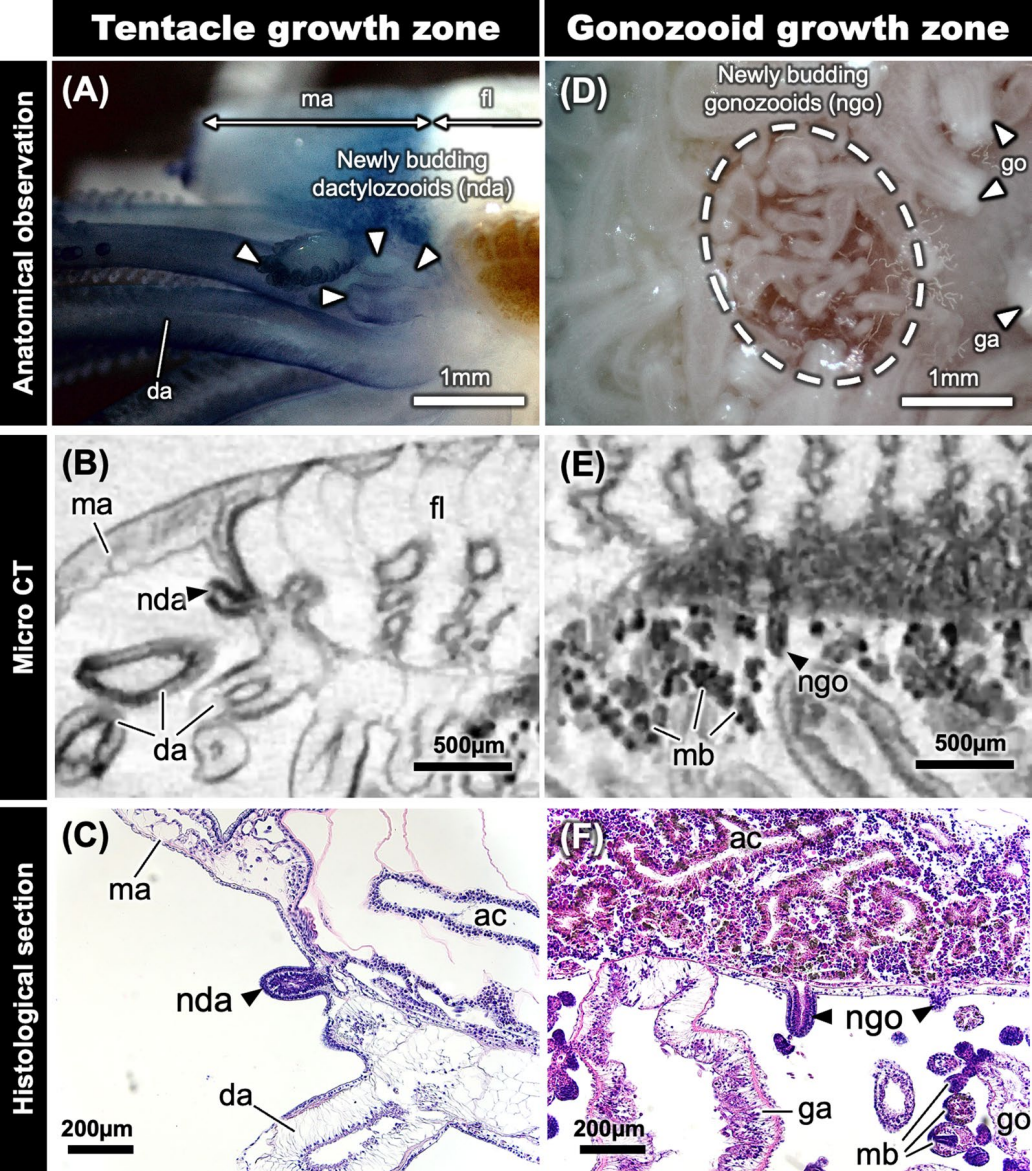
Growth zone of gonozooids and dactylozooids. Gonozooid growth zone (**a**) and dactylozooid growth zone (**d**) as observed in dissected tissue by anatomical survey. Further micro-CT analysis reveals immature gonozooids and dactylozooids within each growth zone (**b**, **e**). Histological sections reveal immature gonozooids and dactylozooids stained darkly with hematoxylin within each growth zone (**c**, **f**). Each structure is shown: mantle (ma), float (fl), newly budding dactylozooid (nda), newly budding gonozooid (ngo), oral chamber (oc), dactylozooid (da), gonozooid (go), gastrozooid (ga), medusa bud (mb)

Next, to clarify the mechanisms by which gonozooids and dactylozooids develop, we examined the distribution of immature gonozooids and dactylozooids (Fig. 4). Brief anatomical observation revealed multiple immature dactylozooids between the mantle and developed dactylozooids (Fig. 4a). Moreover, pouch-like immature dactylozooids structures were also observed in this area, and these were strongly stained with hematoxylin (Fig. 4b, c). On the other hand, immature gonozooids were also observed in great numbers around developed gonozooids (Fig. 4d). Further histological observation also revealed projections of the epithelial cell layer of the coenosarc; these immature gonozooids were also deeply stained with hematoxylin (Fig. 4e, f).

### Development of dactylozooids and gonozooids

**Fig. 5:**
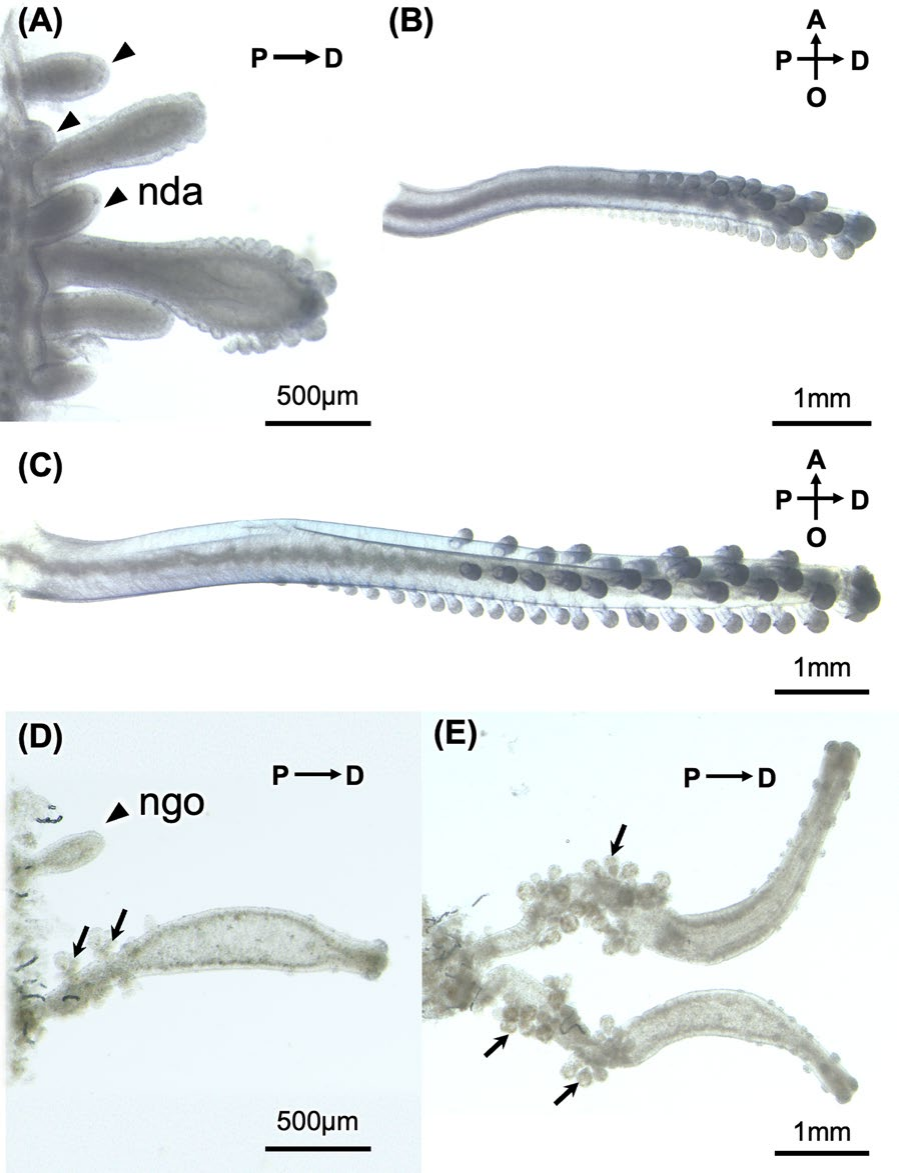
Several developmental stages of dactylozooids (**a**, **b**, **c**) and gonozooids (**d**, **e**). Immature dactylozooids in dactylozooids growth zone. Newly budding dactylozooids (nda: arrowheads) can be seen. Relatively developed dactylozooids have several projections at the distal side of zooids (**b**, **c**). Immature gonozooids at the epithelial of coenosarc (**d**). Relatively developed gonozooids have medusa buds at proximal side and small projections at distal side (**e**)

At the budding areas of the dactylozooids and gonozooids, several developmental stages of each zooid were observed (Fig. 5). Immature dactylozooids, which appeared to have recently emerged, exhibited a simple sac-like structure composed of an epithelial cell layer (Figs. 4b, c, 5a). As they developed, three rows of projections were formed on the distal side of the dactylozooids, with two rows on the lateral side and one on the oral side (Fig. 5c, b), and the number of projections on the oral side was approximately twice that on the lateral side (aboral side: 26 ± 4.1; lateral side: 13.2 ± 2.4; *n* = 9). Similarly to the dactylozooids immediately after budding, immature gonozooids also exhibited a simple sac-like structure composed of an epithelial cell layer (Figs. 4e, f, 5d). In more developed gonozooids, medusa buds were observed at the proximal region, and projections were seen at the distal side, although they were much smaller compared to the projections at the distal side of dactylozooids (Fig. 5d, e).

### Heteromorphic colonies of *P. porpita*

**Fig. 6:**
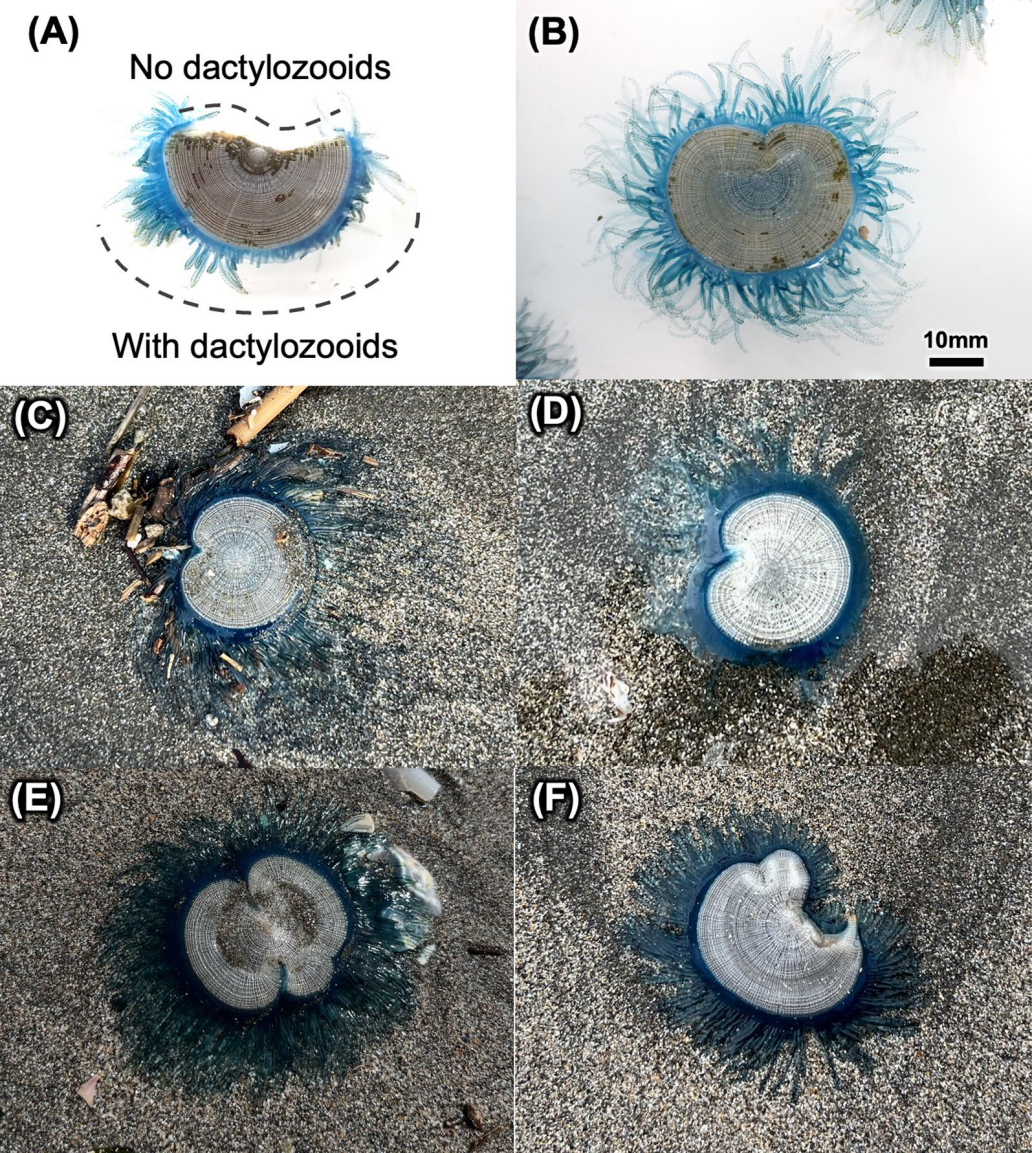
Heteromorphic colonies observed in 2022 (**a**, **b**) and 2024 (**c**, **d**, **e**, **f**). These specimens include one colony that is completely split in half (**a**) as well as a colony that has presumably regenerated (**b**, **c**, **d**). Colonies with large curved margins were also observed (**e**, **f**)

Interestingly, our surveys with citizen sampling also revealed several heteromorphic colonies of *P. porpita* that were not circular but were rather deformed in shape (Fig. 6). Such heteromorphic colonies were observed with a frequency of about five individuals out of more than 200 observations. Almost all of the various deformed colonies had a single gastrozooid, but they differed in the presence or absence of dactylozooids at the margins. Some of these heteromorphic colonies lacked dactylozooids, resulting in exposed internal structures of the coenosarc (Fig. 6a). In contrast, other colonies retained dactylozooids even in the deformed regions, and the float's chamber pattern remained continuous without exposing the internal structures of coenosarc (Fig. 6b–f).

## Discussion

**Fig. 7:**
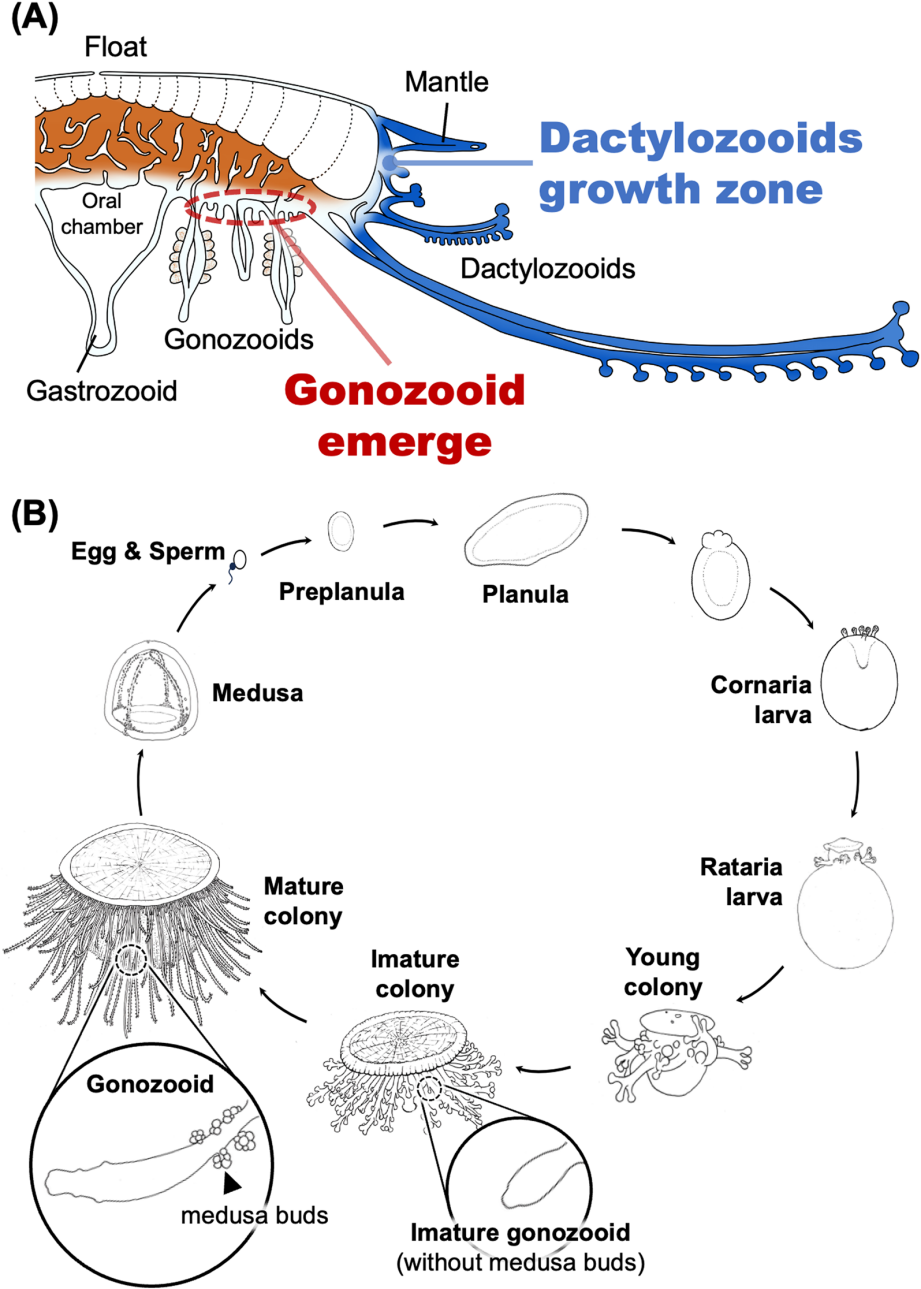
Schematic diagram showing a whole colony including the gonozooid and dactylozooid growth zones **(a)**. Dactylozooid growth zones are located along the margins of the colony, especially at the base of the mantle, while gonozooid growth zones are distributed within the epithelium of the coenosarc. The predicted life cycle and colony development of *Porpita porpita* are also shown **(b)**

The morphological investigations reported in this study revealed that colonies of *P. porpita* can be found in various developmental stages, and range from ~ 0.58 to 38.53 mm in diameter (Fig. 1). The number of gonozooids and dactylozooids tends to increase in proportion to the diameter of the float, and further observation indicated that the number of gonozooids and dactylozooids increased as the colony developed (Fig. 2). In particular, we found that the length of dactylozooids eventually reaches about half the diameter of the float. This dactylozooid length might be optimal for efficiently transporting prey captured at its tip to the mouth of the centrally located gonozooid. In addition, we also found that the number of gonozooids and dactylozooids differed, with gonozooids tending to increase in number more than dactylozooids as the colony developed (Fig. 2c). This difference might be attributed to the different locations where these two structures appear: dactylozooids emerge from the boundary between the mantle and coenosarc, whereas gonozooids emerge randomly from the epithelium of the coenosarc (Figs. 4, 5, 7a). In other words, dactylozooids emerge one-dimensionally along the circumference of the circle, while gonozooids emerge more broadly, in two-dimensional spread all over the face of the circle (Fig. 7a). In fact, increases in the numbers of gonozooids and dactylozooids seemed to follow the ratio of the circumference to the area of the float. Taken together, these results suggest that *P. porpita* has a specific growth zone for dactylozooid which is located at the boundary of the mantle and coenosarc (i.e., outer circumference of the colony) (Figs. 4a–c, 5a, 7a). In addition, gonozooids emerge along the epithelium of the coenosarc between gastrozooid and dactylozooid (Figs. 4d–f, 5d). Particularly, the observation of numerous immature gonozooids surrounding the base of existing gonozooids indicates that the budding region is not restricted but is instead broadly distributed over the epithelium of the coenosarc (Figs. 4d–f, 5d). These findings indicates that, unlike the localized growth zone in dactylozooids, gonozooids do not have a specific growth zone. Rather, the entire coenosarc epithelium between dactylozooids and gastrozooids may function as the growth zone (Fig. 7a).

The formation of sophisticated colonies observed in *P. porpita* and *V. velella* is thought to have evolved within the Capitata lineage [12]. Many species of Capitata, excluding *P. porpita* and *V. velella*, exhibit benthic or epibiotic asexual generations, in which zooids are connected by stolons and form relatively disordered colonies compared to *P. porpita* and *V. velella* [20]. The colony of *V. velella* differs from that of *P. porpita* in being elliptical in shape and possessing a sail, but the arrangement of zooids is similar [15]. Therefore, it is likely that budding zones similar to those found in *P. porpita*—namely, the dactylozooid growth zone located beneath the mantle at the colony periphery, and the budding zone of gonozooids spreading over the epidermis of the coenosarc between gastrozooid and dactylozooids (Fig. 7a)—are also present in *V. velella*. Thus, the spatial arrangement of functionally distinct zooids, and the acquisition of regulatory mechanisms that determine the budding zones responsible for that arrangement, were likely crucial for the evolution of organized colonial structures.

Although phylogenetically distinct, *Physalia physalis*, a siphonophore, also exhibits a pleustonic lifestyle and inhabits the ocean surface, similar to *P. porpita* and *V. velella* [12, 18]. Despite ecological similarities, the colony morphology of *P. physalis* differs significantly in that it lacks a chitinous float like *P. porpita*, and possesses multiple gastrozooids [30, 31]. Therefore, the mechanisms that determine the budding zones and evolutionary processes of colony formation are likely to differ between Porpitidae and Siphonophore.

The formation of a colony that behaves like a single individual has evolved multiple times in Hydrozoa [1, 11, 12, 32]. In siphonophores, the center of the colony shows a linearly extending stem on which two growth zones are localized within a concentrated number of stem cells [21, 22]. As described above, *P. porpita* colonies also show two budding regions, but unlike most siphonophores, they do not have a stem, and their budding regions are spread over the circumference of the circle. This morphological difference suggests that the mechanism of colony formation in these organisms may significantly differ. Furthermore, based on the histological observations made during this study, we report the observation of large round cells stained darkly with hematoxylin within the epithelial cell layer of the inner tubules found in the aboral chamber (Additional file 2: Supplemental Figure S1). These cells morphologically resemble the interstitial stem cells (i-cells) found in hydrozoans [21, 22]; which have a distinct round or spindle shape, and a high nuclear-cytoplasmic ratio [33]. In *P. porpita*, the aboral chambers extend in a complex pattern from the central gastrozooid into the coenosarc, and these chambers may be responsible for nutrition and stem cell supply, as are stolons in ancestral Hydrozoa. Further studies on the localization of stem cell markers such as *vasa, piwi*, and *nanos* in these colonies may further reveal the relationship between stem cell localization and growth zones in this novel clade.

Furthermore, our surveys and citizen samples resulted in the identification of several interestingly heteromorphic colonies of *P. porpita*. These colonies had one gastrozooid and several gonozooids and dactylozooids, just as normal colonies did, but showed margins that were highly deformed. In addition, we also discovered a partially missing dactylozooids colony (Fig. 6a). Compared to these colonies, some of the heteromorphic colonies exhibited a distribution of dactylozooids along the colony margin similar to that observed in normal circular colonies (Fig. 6b–e). This observation suggests that the colony was partially disrupted by an external factor and underwent regeneration afterward. Although it is impossible to know what events happened to produce these heteromorphic colonies, we assume that these colonies result from original colonies being broken up by rocks or reveal predation from sea turtles or other predators; in such cases, damage may trigger the formation of heteromorphic colonies as they undergo a regeneration process. As mentioned above, stem cell like cells were also found in *P. porpita* colonies (Additional file 2: Supplemental Figure S1). It has been suggested that regeneration in metazoans is driven by multiple mechanisms, including cell proliferation and differentiation from stem cells as well as subsequent cellular dedifferentiation, and the regenerative ability of *P. porpita* may involve the stem cells found scattered throughout the coenosarc.

*P. porpita* has not been successfully cultured in the lab for long periods of time, so much of its life history remains unknown. Recently, planula larvae of *P. porpita* have been found [34]. By combining this finding with previous descriptions of larval morphology [13, 32, 35, 36] and our knowledge of colony development, we were able to estimate an approximate life history for *P. porpita* (Fig. 7b). Although eggs and sperm have yet to be identified, after fertilization they likely develop into preplanula and planular larvae. Next, metamorphosis into the “cornaria larvae” stage occurs, followed by the “ratalia larvae” stage during which the float is fully formed. After this stage the larva can grow into a colony. In colonies with a float diameter of less than 10 mm, no medusa formation is observed in the gonozooids; however, when they grow to 10 mm or more, sexual maturation occurs and numerous medusae are produced. The released medusae then reproduce sexually by releasing sperm and eggs to establish the next generation.

## Conclusion

The findings reported in this study reveal that: (1) the micro-CT technique is as effective for revealing colony structure in *P. porpita* as histological sections are; (2) the number and size of gonozooids and dactylozooids increase linearly during colony development; (3) the growth zones of gonozooids and dactylozooids are distributed along the circumference and across the surface of the colony, respectively; (4) the relationship between the growth rates of gonozooids and dactylozooids is approximately proportional to the ratio of the circumference and area of the circle; (5) we observed rare heteromorphic colonies, suggesting that this species has a high regenerative capacity. Taken together, these findings provide valuable insight into the ecology and life history of *P. porpita*.

## Supplementary Information

**Additional file1**. Table S1.Sample preparation and scanning protocol for each specimen)

**Additional file2**. Fig. S1 Horizontal cross-sectional image of the colony via tissue section (A, B). Histological section of the entire colony (A) and magnified image (B). The arrowhead indicates large round hematoxylin-stained cells, which are putative i-cells and arrow shows nematocysts

**Additional file3**. Video S. Continuous sagittal optical section of a colony as obtained by micro-CT.

**Additional file4**. Video S2 3D-constructed video of various colony sizes as obtained by micro-CT

**Additional file5**. Video S3 3D construction image of the gastrozooid as obtained by micro-CT.

**Additional file6**. Fig. S2 3D construction image of the gastrozooid. Side view (A), side view rotated 90 degrees (B), aboral view (C). oral view (D). Arrow indicates aboral holes.

**Additional file7**. Fig. S3 Cross-sectional view of the colony (A). Image with a partially peeled epithelial layer (B). 3D constructed image of chamber with epithelial layer removed (C).

**Additional file8**. Fig. S4 Horizontal micro-CT section of different plane of sectioning, oral (C) and aboral side (D). The central chamber is receding toward the marginal chamber (arrowhead).

**Additional file9**. Video S4 Continuous horizontal optical section around center area of the colony as obtained by micro-CT

**Additional file10**. Fig.S5 Optical cross sections by micro-CT of gonozooid (A) and dactylozooid (B). The aboral chamber is receding toward each structure (arrowhead).

## Data Availability

All data generated or analyzed during this study are included in this published article and its supplementary information files.

## Abbreviations

HE: Hematoxylin and eosin
micro-CT: Microfocus X-ray computed tomography
