# Correction: Zooid arrangement and colony growth in *Porpita porpita*

**DOI:** 10.1186/s12983-025-00568-0

**Published:** 2025-07-14

**Authors:** Kohei Oguchi, Akiteru Maeno, Keita Yoshida, Gaku Yamamoto, Hisanori Kohtsuka, Casey W. Dunn

**Affiliations:** 1 Misaki Marine Biological Station, The University of Tokyo, Miura, Kanagawa 238‑0225 Japan; 2 Department of Biology, Undergraduate School of Biological Sciences, Tokai University, Sapporo, Hokkaido 005‑8601 Japan; 3 Cell Architecture Laboratory, National Institute of Genetics, Yata 1111, Mishima, Shizuoka 411‑8540 Japan; 4 Enoshima Aquarium, Katasekaigan, Fujisawa, Kanagawa 251‑0035 Japan; 5 Department of Ecology and Evolutionary Biology, Curator of Invertebrate Zoology, Yale University, 170 Whitney Ave, Peabody Museum New Haven, CT 06511 USA

**Correction: Frontiers in Zoology (2025) 22:11** 10.1186/s12983-025-00565-3

Following publication of the original article [1], author reported the captions to Figs. 5 and 6 were swapped. Captions have been revised to:

**Fig. 5:**
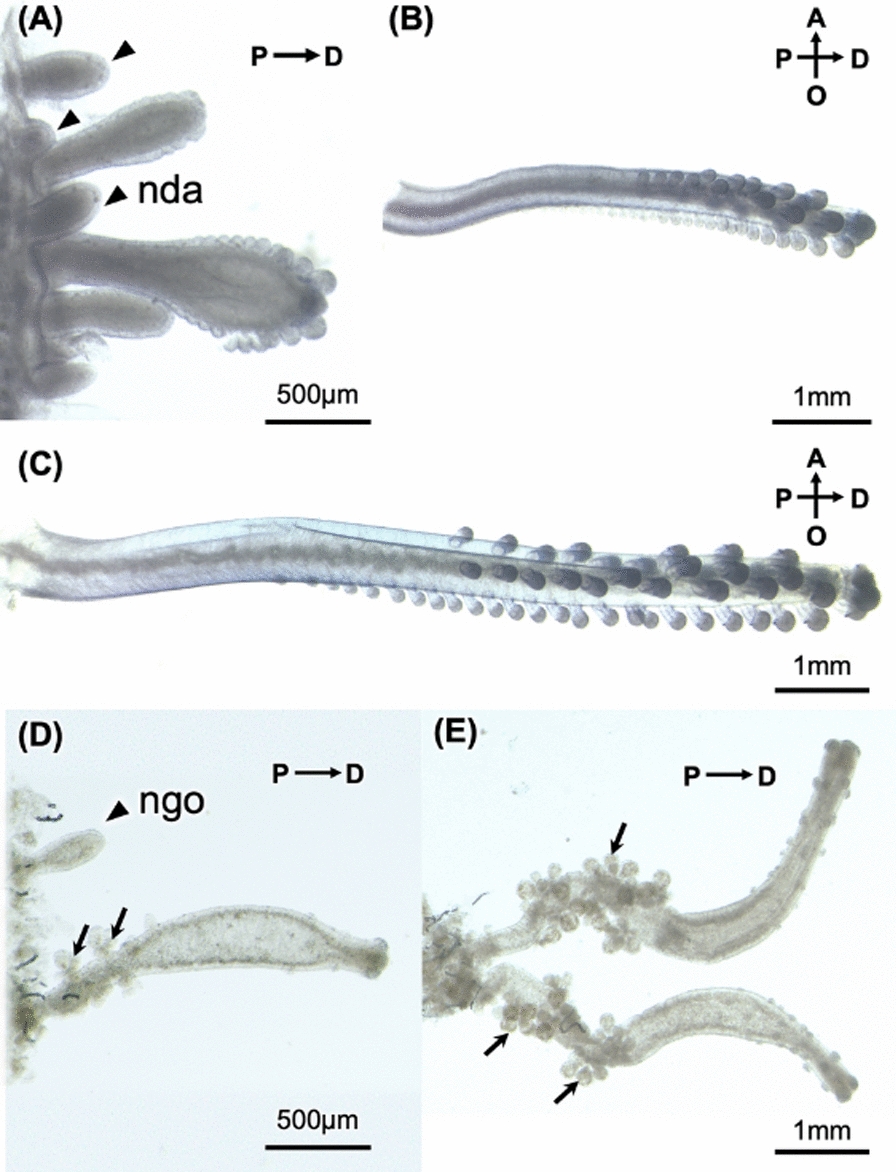
Several developmental stages of dactylozooids (**a, b, c**) and gonozooids (**d, e**). Immature dactylozooids in dactylozooids growth zone. Newly budding dactylozooids (nda: arrowheads) can be seen. Relatively developed dactylozooids have several projections at the distal side of zooids (**b, c**). Immature gonozooids at the epithelial of coenosarc (**d**). Relatively developed gonozooids have medusa buds at proximal side and small projections at distal side (**e**)

**Fig. 6:**
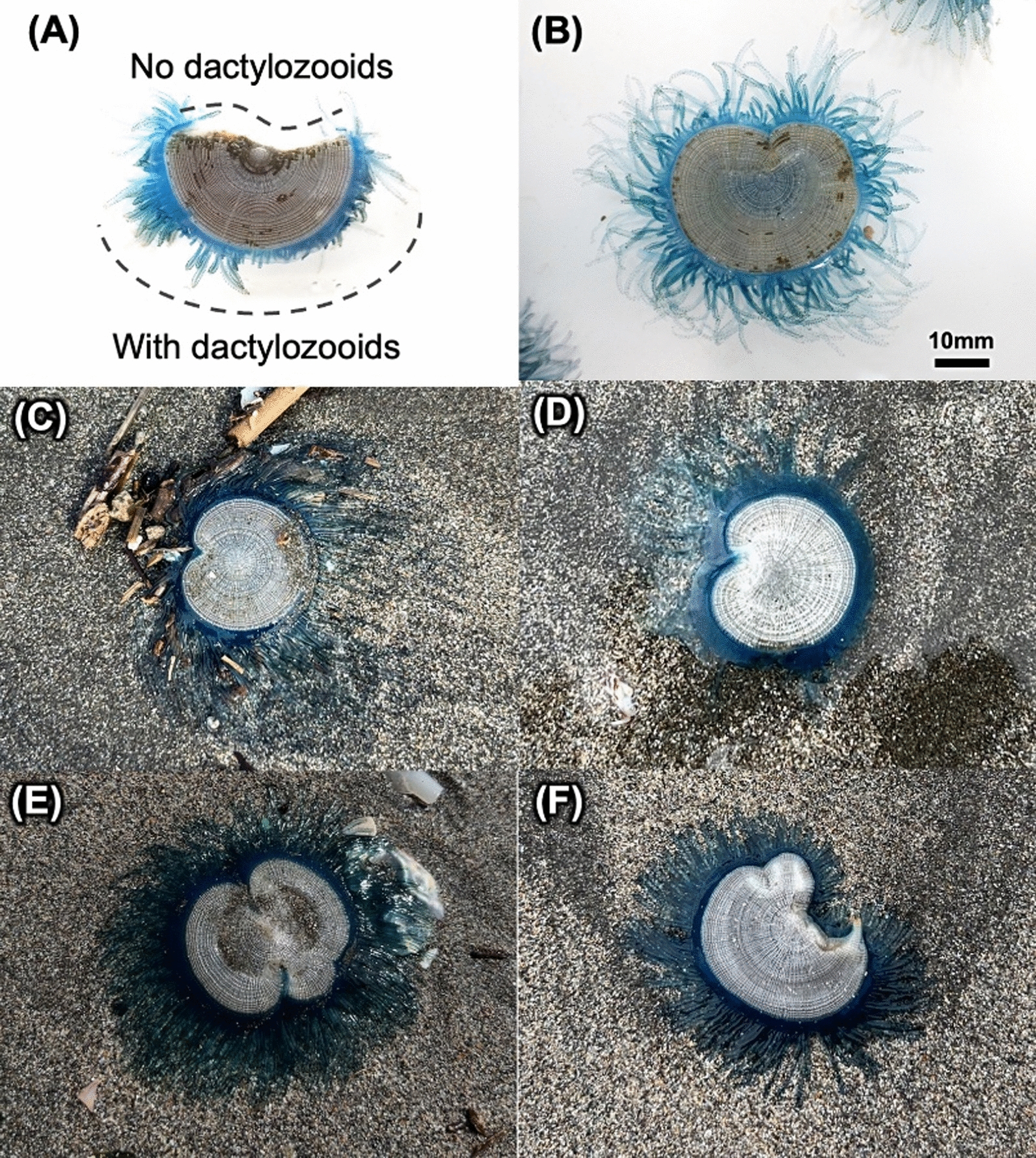
Heteromorphic colonies observed in 2022 (**a, b**) and 2024 (**c, d, e, f**). These specimens include one colony that is completely split in half (**a**) as well as a colony that has presumably regenerated (**b, c, d**). Colonies with large curved margins were also observed (**e, f**)

The original article [1] has been updated.
