# Supplementary figures and images for: Zooid arrangement and colony growth in Porpita porpita
Source: Front Zool. 2025 Jun 23;22:11. doi: 10.1186/s12983-025-00565-3 (PMC12183843; doi:10.1186/s12983-025-00565-3)

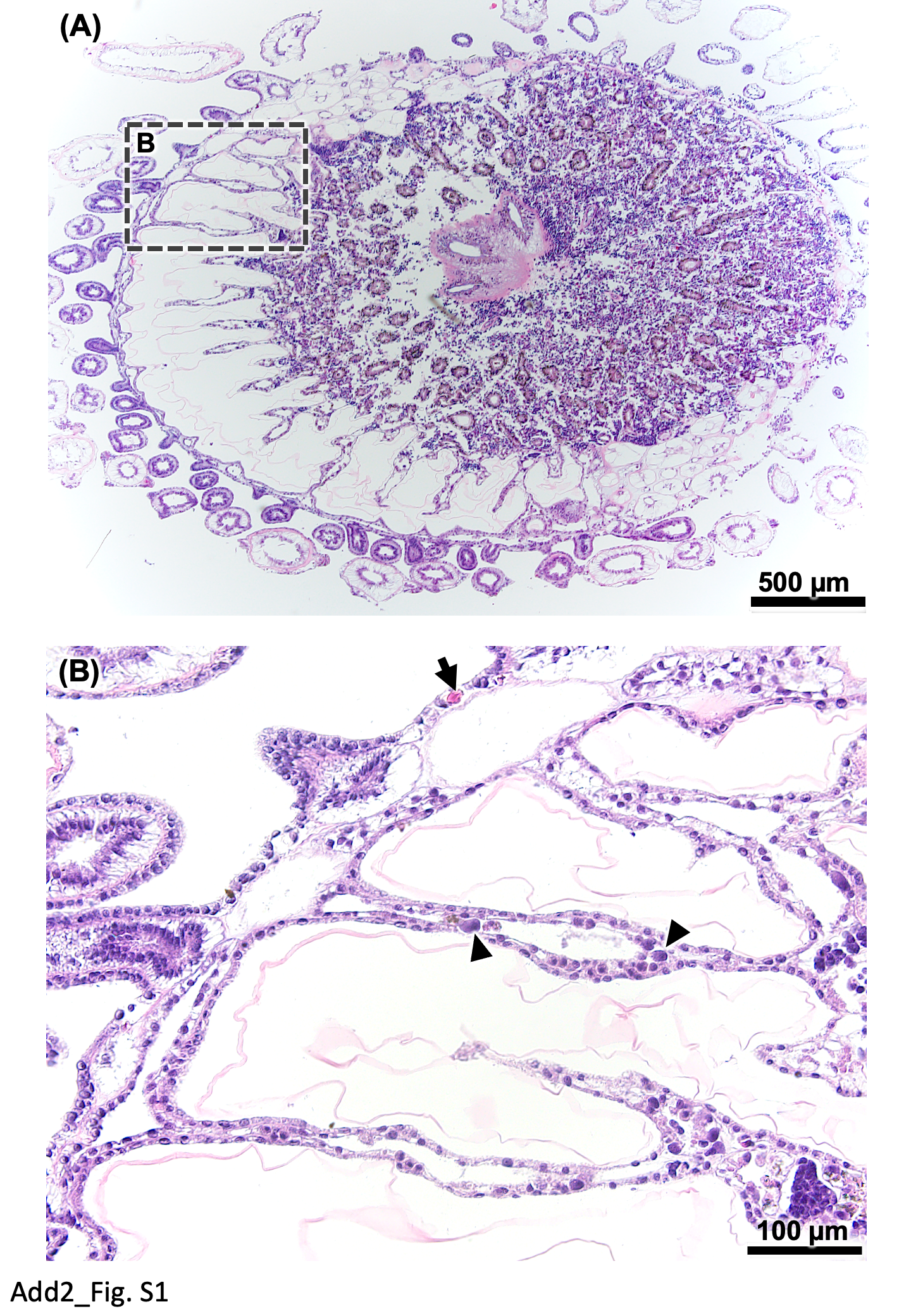

Supplement: Supplementary file 2 — Additional file2. Fig. S1 Horizontal cross-sectional image of the colony via tissue section (A, B). Histological section of the entire colony (A) and magnified image (B). The arrowhead indicates large round hematoxylin-stained cells, which are putative i-cells and arrow shows nematocysts [file 12983_2025_565_MOESM2_ESM.tiff]

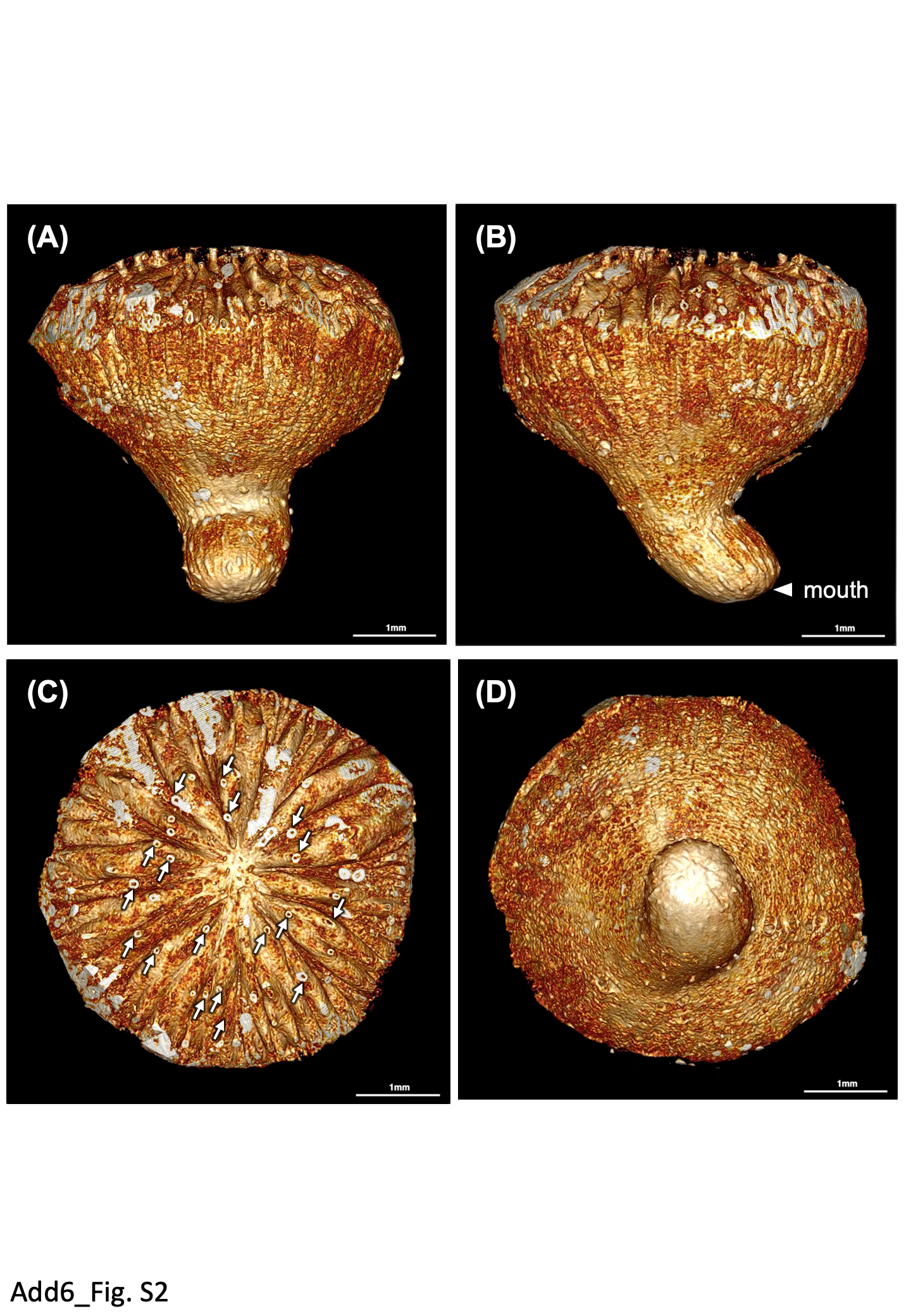

Supplement: Supplementary file 6 — Additional file6. Fig. S2 3D construction image of the gastrozooid. Side view (A), side view rotated 90 degrees (B), aboral view (C). oral view (D). Arrow indicates aboral holes. [file 12983_2025_565_MOESM6_ESM.tiff]

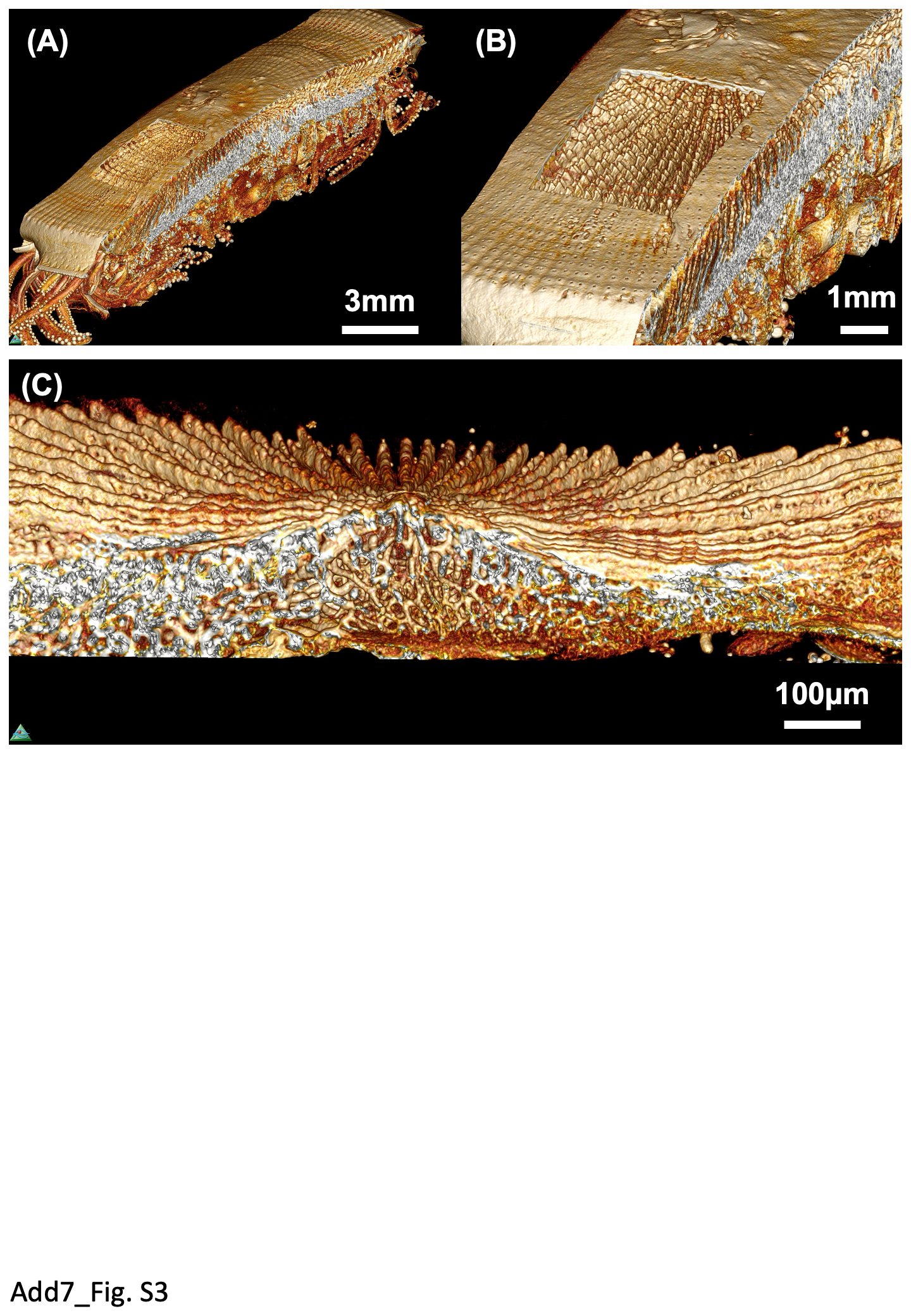

Supplement: Supplementary file 7 — Additional file7. Fig. S3 Cross-sectional view of the colony (A). Image with a partially peeled epithelial layer (B). 3D constructed image of chamber with epithelial layer removed (C). [file 12983_2025_565_MOESM7_ESM.tiff]

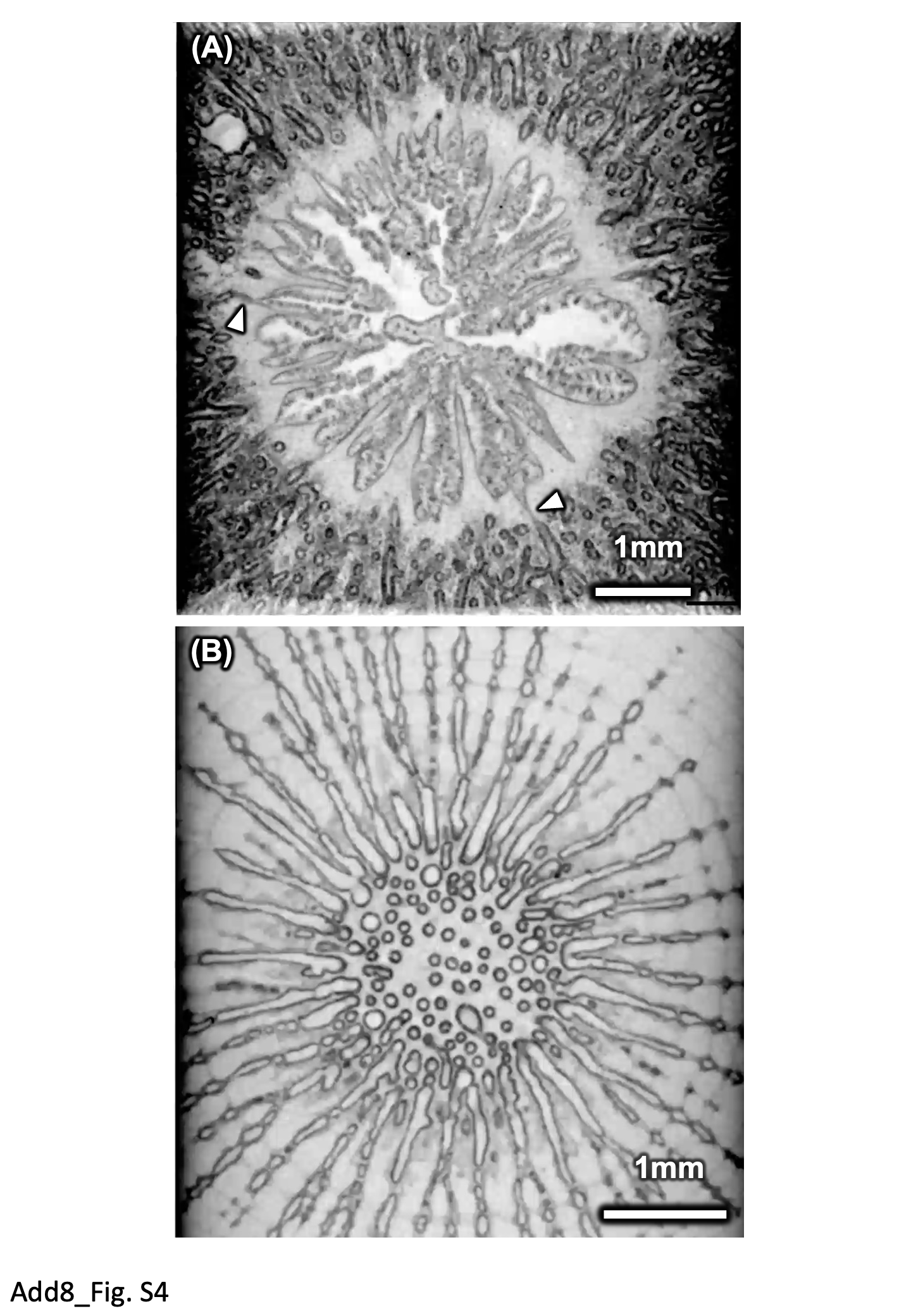

Supplement: Supplementary file 8 — Additional file8. Fig. S4 Horizontal micro-CT section of different plane of sectioning, oral (C) and aboral side (D). The central chamber is receding toward the marginal chamber (arrowhead). [file 12983_2025_565_MOESM8_ESM.tiff]

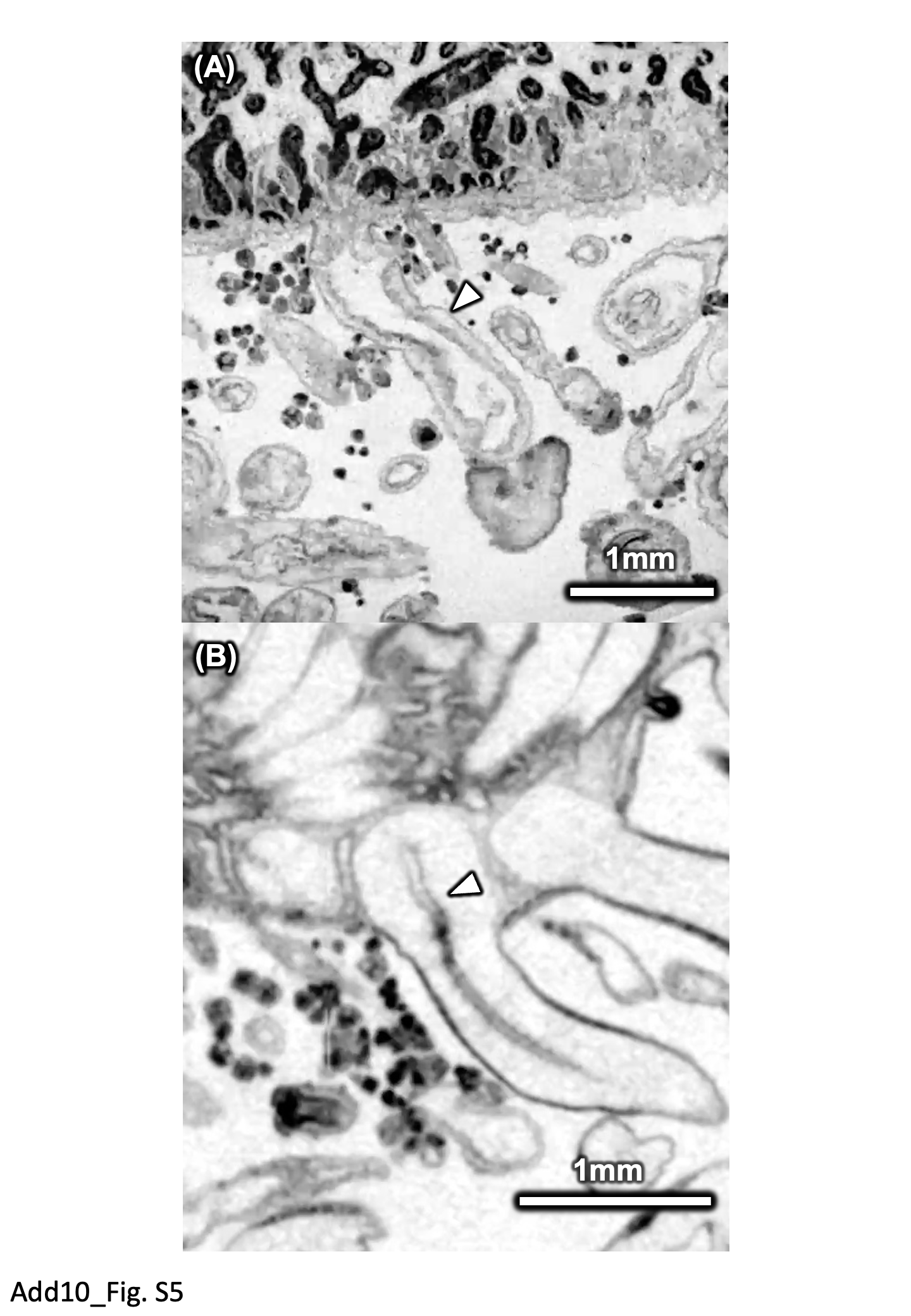

Supplement: Supplementary file 10 — Additional file10. Fig.S5 Optical cross sections by micro-CT of gonozooid (A) and dactylozooid (B). The aboral chamber is receding toward each structure (arrowhead). [file 12983_2025_565_MOESM10_ESM.tiff]
